# Pharmaceuticals and personal care products’ (PPCPs) impact on enriched nitrifying cultures

**DOI:** 10.1007/s11356-021-14696-7

**Published:** 2021-06-24

**Authors:** Carla Lopez, Mac-Anthony Nnorom, Yiu Fai Tsang, Charles W. Knapp

**Affiliations:** 1 Centre for Water, Environment, Sustainability & Public Health, Department of Civil & Environmental Engineering, University of Strathclyde, Glasgow, G1 1XJ UK; 2 Department of Science and Environmental Studies, The Education University of Hong Kong, Tai Po, New Territories 999077 Hong Kong

**Keywords:** PPCPs, Nitrifying bacteria, Nitrification inhibition, Acute toxicity, Nitrogen removal

## Abstract

The impact of pharmaceutical and personal care products (PPCPs) on the performance of biological wastewater treatment plants (WWTPs) has been widely studied using whole-community approaches. These contaminants affect the capacity of microbial communities to transform nutrients; however, most have neither honed their examination on the nitrifying communities directly nor considered the impact on individual populations. In this study, six PPCPs commonly found in WWTPs, including a stimulant (caffeine), an antimicrobial agent (triclosan), an insect repellent ingredient (*N*,*N*-diethyl-*m*-toluamide (DEET)) and antibiotics (ampicillin, colistin and ofloxacin), were selected to assess their short-term toxic effect on enriched nitrifying cultures: *Nitrosomonas* sp. and *Nitrobacter* sp. The results showed that triclosan exhibited the greatest inhibition on nitrification with EC_50_ of 89.1 μg L^−1^. From the selected antibiotics, colistin significantly affected the overall nitrification with the lowest EC_50_ of 1 mg L^−1^, and a more pronounced inhibitory effect on ammonia-oxidizing bacteria (AOB) compared to nitrite-oxidizing bacteria (NOB). The EC_50_ of ampicillin and ofloxacin was 23.7 and 12.7 mg L^−1^, respectively. Additionally, experimental data suggested that nitrifying bacteria were insensitive to the presence of caffeine. In the case of DEET, moderate inhibition of nitrification (<40%) was observed at 10 mg L^−1^. These findings contribute to the understanding of the response of nitrifying communities in presence of PPCPs, which play an essential role in biological nitrification in WWTPs. Knowing specific community responses helps develop mitigation measures to improve system resilience.

**Supplementary Information:**

The online version contains supplementary material available at 10.1007/s11356-021-14696-7.

## Introduction

Recently, the widespread presence of pharmaceutical and personal care products (PPCPs) in the environment has drawn the attention of the research community due to the potential adverse effects on ecosystems and human health (Kümmerer 2009; Tran et al. 2018). Studies on the occurrence and fate of PPCPs have shown that stimulants, antimicrobial agents, repellents and antibiotics have frequently been detected in aquatic systems and engineered facilities such as wastewater treatment plants (WWTPs). These compounds, representing a wide range of human consumption products disposed and excreted into the sewage systems, pass through WWTPs and are discharged into the water bodies (Balakrishna et al. 2017; Yang et al. 2017).

Among the PPCPs, caffeine (CF) is one of the most abundant chemicals in WWTP samples, and its concentrations range from a few ng L^−1^ to μg L^−1^ (Luo et al. 2014; Tran et al. 2014; Balakrishna et al. 2017; Paíga et al. 2019). CF is an organic stimulant compound that is commonly added to beverages and other products. Although the literature shows that CF is highly biodegradable in biological WWTPs with removal efficiencies >80% (Sui et al. 2010; Dai et al. 2014; Tran et al. 2018), the increase in CF consumption worldwide may lead to higher amounts discharged to the water bodies, increasing the risk of exposure in the ecosystems (Quadra et al. 2020).

Other substances frequently detected in WWTPs include the antimicrobial agent triclosan (TCS) and the active ingredient of insect repellent *N*,*N*-diethyl-*m*-toluamide (DEET) (Liu and Wong 2013; Yang et al. 2017; Juksu et al. 2019). TCS concentrations have been reported in different influents of WWTPs worldwide, and the values are as high as 0.47 μg L^−1^ in China (Zheng et al. 2020), 86.1 μg L^−1^ in the USA (Kumar et al. 2010) and 17.6 μg L^−1^ in South Africa (Lehutso et al. 2017). In the case of DEET, the concentrations observed in WWTPs range from a few ng L^−1^ up to peak values as high as 15.8 μg L^−1^ in Europe (Merel and Snyder 2016) and 42.3 μg L^−1^ in the USA during the summer season (Mohapatra et al. 2016). Due to the variable removal efficiencies in WWTPs (Luo et al. 2014), TCS and DEET can be found in different environmental matrices, such as treated effluent, surface waters, waste sludge and sediments (Ramaswamy et al. 2011; Dai et al. 2014; Zhao et al. 2010; Dsikowitzky et al. 2020).

One of the most concerning pharmaceuticals in WWTPs are antibiotic residues. These compounds treat different infectious diseases and their disposal into the environment toxicologically impacts non-target microorganisms in the ecosystems, develops antimicrobial resistance, and contaminates soils and water bodies (Kümmerer 2009). Studies have shown that many antibiotic compounds have often been excreted in urine and/or faeces, cleansed off bodies or even disposed directly to sewers with minimal change after their administration (Marx et al. 2015); they are frequently detected in influent and effluent on WWTPs, suggesting a degree of persistence through treatment plants (Tran et al. 2016; Mutiyar and Mittal 2014; Leung et al. 2012). Survey-based studies indicate that conventional WWTPs generally do not efficiently remove antibiotics (Paíga et al. 2019)

According to the review of antimicrobial consumption by Robertson et al. (2019), β-lactams were the most commonly prescribed antibiotics worldwide in 2015. In this antibiotic class, ampicillin (AMP) has been widely used in human medicine and is considered highly degradable due to its unstable β-lactam ring structure (Watkinson et al. 2007). However, the chemical transformation of β-lactam antibiotics could vary depending on the matrix conditions (Mitchell et al. 2014), where in some cases AMP could still be detected even in treated effluent from WWTPs (Mutiyar and Mittal 2014).

Other predominant antibiotics in WWTPs are fluoroquinolones (Tran et al. 2018). Within this group, ofloxacin (OFX) is a second-generation antibiotic applied to treat urinary tract infections (King et al. 2000). Although restricted by the WHO (Robertson et al. 2019), the presence of OFX continues in raw sewage and effluent in WWTPs (Brown et al. 2006; Dinh et al. 2017), reaching concentrations of 7.9 μg L^−1^ in Asia (Leung et al. 2012; Minh et al. 2009) and 8.6 μg L^−1^ in Europe (Dinh et al. 2017).

Another source of antibiotics in WWTPs are veterinary medicines (Kemper 2008). Under this application, colistin (CST) is a polymyxin antibiotic that is widely used in animal farms to treat Gram-negative infections (Liu et al. 2016; Kempf et al. 2016) and it has re-emerged as a ‘last-resort’ antibiotic to target multidrug-resistant infections (Dagla et al. 2019). The occurrence of CST remains limited given that analytical methods for its quantification remain under development for environmental samples (Song et al. 2020). However, the detection of CST on biological matrices (Dagla et al. 2019) and the presence of a CST resistome in bacteria from WWTPs (Hembach et al. 2017) suggest that CST may pose a risk to microbial communities.

The presence of PPCPs in WWTPs is crucial because they can adversely affect biological treatment processes; these systems rely on microbial communities to transform nutrients, such as nitrogen, to prevent aquatic eutrophication (Xiao et al. 2015). For example, biological nitrification, which is part of the nitrification–denitrification reaction sequence in WWTPs, involves a two-step process carried out by two autotrophic microorganisms, namely ammonia-oxidising bacteria (AOB) and nitrite-oxidising bacteria (NOB) (Koops and Pommerening-Röser 2001). Moreover, the performance of AOB–NOB communities can be disrupted due to their fragile mutualism (Graham et al. 2007; Knapp and Graham 2007), low phylogenetic diversity, slow growth characteristics and sensitivity to toxic chemicals (Li et al. 2016).

The role of nitrifiers on biological nitrogen removal is a critical process in wastewater treatment and their response against toxic chemicals is of great concern for the stability and performance of WWTPs (Xiao et al. 2015). The adverse effect of pharmaceuticals on nitrifying communities has been reported in wide range of conditions including short- and long-term exposure at different concentrations. The findings have shown that these compounds can decrease nitrification rates, leading to poor ammonium removal efficiency and disruption of the AOB–NOB mutual cooperation, producing partial nitrification with nitrite accumulation. Other effects observed are the inhibition of enzymatic activities of AOB–NOB species with reduction of ammonia-monooxygenase (AMO) and nitrite-oxidoreductase (NOR) enzymes and the variation of the bacterial community composition, shifting their richness and diversity (Kong et al. 2017; Yu et al. 2019; Li et al. 2020; Zhang et al. 2020).

Despite published data, toxicity assessments of the common PPCPs on AOB–NOB communities are limited and even unavailable in some cases. Most of the studies found in literature were performed with high concentrations of activated sludge as the biomass source, where the presence of more diverse microbial populations and high solid content could lead to varied inhibition results on nitrifying species (Lakshminarasimman et al. 2018; Armstrong et al. 2019; Zhang et al. 2020).

This study investigated the effect of selected PPCPs, including a stimulant (CF), personal care products (DEET and TCS) and antibiotics (AMP, OFX and CST), on an enriched nitrifying community. Batch reactors were employed to assess the acute toxicity of these substances, where changes of ammonium, nitrite and nitrate concentrations were measured to determine nitrification inhibition. Enriched nitrifying bacteria were selected as the inoculum with efforts to increase nitrification activity, control the presence of heterotrophic bacteria and remove solids from activated sludge, which could alter nutrients, transform the toxic substances and interfere with the interaction of nitrifying bacteria with the test substance (Zhang et al. 2020). The findings obtained in this study expand our understanding of the short-term effects of PPCP exposure on nitrifying bacteria, which could importantly prevent the failure of biological nitrogen removal systems in WWTPs.

## Materials and methods

Cultivation of nitrifying bacteria

Activated sludge, collected from a WWTP in Scotland, was used as the source of nitrifying bacteria. The enrichment of nitrifiers was carried out in batch cultures according to the procedure described by Bollmann et al. (2011) and Radniecki and Lauchnor (2011). The nutrient media were modified from Bollmann et al. (2011) with the following final chemical composition (g L^−1^): 0.5 (NH_4_)_2_SO_4_ as an inorganic nitrogen source; 0.585 NaCl, 0.054 KH_2_PO_4_, 0.147 CaCl_2_∙H_2_O, 0.075 KCl, 0.049 MgSO_4_∙7H_2_O and 0.5 NaHCO_3_ as the inorganic carbon source; 7.21 HEPES as a buffer and 1 mL of trace elements solution from Schmidt and Belser (1994). After autoclaving at 121°C for 20 min, the pH media was adjusted to 7.6 ± 0.2 (pH/conductivity meter; Mettler Toledo, MPC 227, Switzerland) with 10 M NaOH (sodium hydroxide solution; Fisher Scientific).

In the first stage of enrichment, 1 g (wet mass) of activated sludge was inoculated into 100-mL Erlenmeyer flasks with media, followed by a series of repeated transfers in fresh media to promote the growth of nitrifiers as explained by Bollmann et al. (2011). The procedure aimed to reduce the activated sludge flocs and particles that could interact or degrade the test substance, and minimise nitrogen assimilation by heterotrophic bacteria that could affect nitrification measurements (ISO 9509 2006; Chen et al. 2014). This process continued for 3 months, where AOB/NOB activities in each culture flask were evaluated through visible observation of ammonium disappearance using Nessler reagent (HACH, Germany), and spots test strips for nitrite and nitrate detection (AquaChek; HACH, Germany).

Once the cultures presented stable activity, the bacterial broth was transferred to 2-L glass bottles (three lab-scale reactors in total) for further enrichment and provide sufficient inoculum for the toxicity tests. The air was supplied with an air pump (HDOM, Model HD-603; Shenzhen Hidom Electric Co., Ltd.) filtered with a 0.2-μm sterilising-grade filter (Aervent™) to maintain the dissolved oxygen (DO) above 4 mg L^−1^ (DO meter; Eutech Instruments Pte Ltd., DO 6+ DO/Temp, Singapore). The reactors were operated at room temperature (20–27°C) and were periodically provided with (NH_4_)_2_SO_4_ solution as substrate and NaOH solution (sodium hydroxide solution 10 M; Fisher Scientific) to maintain optimum pH (7.6–7.8). The reactors’ working volume was 1.7 L and every 2 weeks, 1.2 L of supernatant was removed and replaced with the same volume of fresh nutrient medium to prevent excessive accumulation of by-products.

Batch toxicity assays

We consulted the ISO 9509 (2006) protocols for the experiment design; it evaluates the exclusive capacity of nitrifiers to transform inorganic nitrogen into oxidation species, and it represents a more sensitive approach (Stasinakis et al. 2008; Yuan et al. 2019; Brandt et al. 2015). Study duration was extended up to 2–3 days in contrast to the few hours proposed by the protocol ISO 9509 (2006), to account for the relatively slow-growing populations (Radniecki and Lauchnor 2011). For each assay, the incubation was finalised before ammonium concentrations reached zero to avoid substrate limitation.

An enriched nitrifying community was selected; it remains representative of the AOB–NOB communities present in WWTPs (Li et al. 2016) but minimises interference of ammonia assimilation by excessive heterotrophic bacteria. The experimental conditions of the batch assays for the six PPCPs are summarised in Table 1. The tests were performed individually in the following order: CF, AMP, TCS, DEET, CST and OFX. Batch cultures were undertaken in 500-mL glass bottles with 300-mL working volume. Each treatment was inoculated with 50 mL (equivalent to 337 ± 19 mg VSS L^−1^) of bacterial suspension harvested from the 2-L (enriched stock) reactors. Due to the slow growth of nitrifying bacteria, the first three tests (CF, AMP and TCS) were run in duplicate to cover a broad range of concentrations. Eventually, sufficient quantities of biomass stock were generated to run the experiments in triplicates for the last three toxicants (DEET, CST and OFX). Similar nutrient media were prepared for the assays with lower initial ammonium concentration (<56 mg L^−1^ NH_4_^+^-N) as recommended by ISO 9509 (2006).

**Table 1:** Initial operating conditions for the short-term batch assays

Variable	CF	AMP	TCS	DEET	CST	OFX
NH_4_^+^-N (mg L^−1^)*	53.3 ± 0.6	53.2 ± 0.6	53.1 ± 0.5	50.3 ± 0.5	50.6 ± 0.7	49.7 ± 0.6
NO_2_^−^-N (mg L^−1^)*	1.0 ± 0.1	1.0 ± 0.1	0.7 ± 0.1	1.5 ± 0.2	0.7 ± 0.2	1.6 ± 0.2
NO_3_^−^-N (mg L^−1^)*	0.8 ± 0.1	0.5 ± 0.2	0.5 ± 0.1	3.7 ± 0.2	4.6 ± 0.5	9.4 ± 0.4
pH range	7.4–7.7	7.8	7.7–7.8	7.6–7.8	7.7	7.4–7.7
Temperature (°C)	20–22	20–22	22–27	20.5–22	21.5–22	21–22
DO (mg L^−1^)	>5	>5	>5	>5	>5	>5
Protein (mg L^−1^)	4.7	4.7	6.3	6.3	6.3	9.0
Replicates	Duplicates	Duplicates	Duplicates	Triplicates	Triplicates	Triplicates
Volume (mL)	300	300	300	300	300	300
Duration (h)	73	68	34	43	74	33

*Nitrogen values are represented by mean ± standard deviations

Before inoculation, the biomass was subjected to a cleaning procedure that involved centrifugation, settling, decanting and resuspension to remove any remaining traces of oxidised products and minimise organic material, and ensure sufficient buffering capacity during the assays (Moussa et al. 2003; Salem et al. 2006). Initially, the nitrifying biomass used for the first three PPCPs (i.e. CF, AMP and TCS) were centrifuged at 10,000×*g* for 35 min, settled (20 min), decanted and refilled with new test media; this procedure was repeated twice. It was observed that longer centrifugation and settling time of the biomass led to significant nitrite accumulation in the batch assays; thus, the washing procedure for the last three PPCPs (i.e. DEET, CST and OFX) was optimised by halving processing times.

During experimentation, cultures were supplied with humidified air via aeration stones at the bottom of each bottle to maintain DO above 4 mg L^−1^. pH was measured at the beginning and end of the incubation period. Temperature was recorded using a USB Temperature Data Logger (Lec, EasyLog USB version 7.6.0.0; Lascar Electronics Ltd.).

Biomass concentrations were estimated by their protein content using the Micro BCA Protein Assay kit (Thermo Scientific, USA), following their procedure. Volatile suspended solids (VSS) concentrations were determined according to Standard Methods (APHA 1998). All batch assays (per test substance) were inoculated with the same amount of biomass and substrate concentration, and the protein concentrations were 6.2 ± 1.6 mg L^−1^ (equivalent to 71 ± 5 mg VSS L^−1^); changes in microbial protein content between the beginning and end of the incubation period were negligible.

As an additional treatment ‘control’ and to compare activity responses of the consortium, reference inhibitor allylthiourea (ATU) was used at 0.3 mg L^−1^, a selective ammonia-monooxygenase inhibitor of AOB populations (Gwak et al. 2020) and nitrification. AOB are considered the limiting step of nitrification (ISO 9509 2006). This was used to verify whether ammonium disappearance was resultant of autotrophic nitrification.

Samples were collected during the incubation period, filtered with a 0.45-μm cellulose filter and preserved following protocol BSI EN ISO 5667-3 (2018) for analysis of ammonium, nitrite and nitrate. Unfiltered samples were collected for DNA and protein test and preserved at −80°C until analysis.

The nitrification activity in the batch assays was measured by monitoring nitrogen species over time. The oxidised nitrogen (NO_X_-N), the sum of NO_2_^−^-N and NO_3_^−^-N concentrations, obtained for the different treatments were used to calculate the inhibition percentages in Eq. (1). The values correspond to the measurements at the end of each experiment and reported as the mean value of triplicate or duplicate assays. These percentages were plotted as a function of the toxicant concentrations.

1 $$ \% Inhibition=\frac{\left({NO}_{X\  control}-{NO}_{X\  test}\right)}{NO_{X\  control}}\times 100 $$

*NO*_*X test*_ and *NO*_*X control*_ represented the concentrations of oxidised nitrogen (mg-N L^−1^) in each batch reactor with the toxic substance, and the ‘control’ absent of the toxicant. All concentrations were adjusted by subtracting the initial concentration of nitrite or nitrate to record the variation of the oxidation species over the incubation period.

The substance concentration that decreases nitrification activity in comparison to the controls by 50% is defined as 50% effective concentration (EC_50_). This value was estimated by interpolating the graph of inhibition percentage (Eq. (1)) against the log-transformed toxicant concentration. The profile was adjusted to a linear or polynomial model, considering the best fit with a coefficient of determination (*R*^2^) >0.96 (ISO 9509 2006).

Test substances

Caffeine (>95% purity), irgasan or triclosan (≥97% purity), *N*,*N*-diethyl-3-methylbenzamide or DEET (>97% purity), ampicillin (ready-made solution, 100 mg mL^−1^), ofloxacin (≥99% purity) and colistin sulphate salt (≥15,000 U mg^−1^) were purchased from Sigma Aldrich. The range of concentrations selected for the batch assays is presented in Table 2. These concentrations were chosen to include a range commonly found in WWTPs based on the values reported in the literature for either WWTPs or previous inhibition studies (see Table 2); however, higher concentrations were included to evaluate whether target compounds would produce any response to nitrification performance (Pasquini et al. 2013).

**Table 2:** PPCP concentrations tested in batch reactors

Class	Substance	CAS number	Concentrations (mg L^−1^)	Reference
Stimulant	CF	58-08-2	0.025, 0.115, 1, 10, 40, 90	(Gheorghe et al. 2016)
Antimicrobial	TCS	3380-34-5	0.01, 0.1, 0.3, 0.5, 1, 2	(Roh et al. 2009)
Insect repellent	DEET	134-62-3	0.02, 0.1, 1, 5, 10	(Aronson et al. 2012)
Antibiotics	AMP	69-52-3	0.5, 5, 50, 100, 175, 250	(Gomez et al. 1996)
	CST	1264-72-8	0.1, 1, 10, 100, 350	(Bressan et al. 2013)
	OFX	82419-36-1	0.01, 0.1, 1, 5, 10	(Dokianakis et al. 2004)

Stock solutions were prepared on the same day of the assay, and Milli-Q water was used for the antibiotics and CF. Because TCS and DEET have poor solubility in water, the solutions were prepared with dimethyl sulphoxide (DMSO) as solvent (<0.1% v/v), and similar concentrations of DMSO were maintained in all treatments.

The fate of the substances was not analysed, but it was considered in the assays; reported half-lives were CF, a few hours (Dorival-García et al. 2013); OFX, >4 days (Dorival-García et al. 2013); DEET, days to weeks (Weeks et al. 2012; Lakshminarasimman et al. 2018); and partial biodegradation reported for TCS (Lakshminarasimman et al. 2018) and AMP (Ramírez Muñoz et al. 2020). Test bottles were covered with foil to prevent light exposure and possible photolysis (e.g. Bedoux et al. 2012); pH and temperature were balanced between microbial activity and compound stability (e.g. Mitchell et al. 2014; Li et al. 2003). Most previous studies used activated sludge with VSS quantities 10^2^–10^3^ times higher than this study. Therefore, it is hoped that low biomass levels and autotrophic nature of the media in the assays minimised degradation of the tested chemicals.

Analytical methods

The concentrations of ammonium, nitrite and nitrate were determined through colorimetric analysis using KoneLab Aqua 30 (Thermo Scientific, Aquarem 300; Clinical Diagnostics Finland) according to the British Standard procedures BS ISO 15923-1 (2013). For the colorimetric analysis, pre-tests involved spiked controls to determine whether any interference by other compounds had any effect on assays. All the reagents were purchased from Thermo Fisher.

DNA extraction and 16S-rRNA gene sequencing and analysis

Four samples were tested to analyse their microbial community structure. Two were collected from the bioreactors at the beginning of the experiments (S1 and S2) and the two other (S3 and S4) at the end of the testing period, after ~8 months of harvesting. All samples were stored at −80 °C in 2-mL tubes prior to the analysis.

DNA were extracted from biomass samples collected from the reactors using a QIAGEN DNeasy® Blood & Tissue Kit, according to the manufacturer’s instructions. The DNA quantity was estimated using a Spectrophotometer Microplate Epoch (BioTek Instruments, Inc., USA) and data collection and analysis software Gen5^TM^ V1.11.5 (BioTek Instruments, Inc., USA).

DNA sequencing was performed at Glasgow Polyomics (Glasgow, UK) using Illumina MiSeq platform, targeting the 16S-rRNA operon for taxonomy, using recommended primers by RDP-II Pipeline (Maidak et al. 2001; https://rdp.cme.msu.edu); and further, bioinformatic identification of microbial community was performed with QIIME2 version 2021.2 (Bolyen et al. 2019), with similar data analysis detailed by Al Ali et al. (2020) (see Supplementary information).

## Results and discussion

During the pre-experimental enrichment process, ammonium conversion to nitrite increased rapidly and low nitrate production was observed (0.3 mg NO_3_^−^-N/mg NH_4_^+^-N consumed), leading to nitrite build-up in the reactors. However, nitrite accumulation gradually decreased, reaching undetectable values after 2 months of operation. Subsequently, the reactors achieved a stable nitrification performance, maintaining an ammonium consumption rate between 11 and 20 mg NH_4_^+^-N/g MLVSS h and a nitrate production yield of 0.95 mg NO_3_^−^-N/mg NH_4_^+^-N—i.e. 95% of ammonia disappearance was attributed to nitrification. These cultures were sustained in batch reactors and were the ‘stock’ for subsequent assays.

Microbial community

The taxonomic classification derived from the 16S rRNA gene sequencing and analysis is illustrated in Fig. S1 (Supplementary information). At phylum level, *Proteobacteria* were dominant in all analysed samples, accounting for 63–68% of the total bacterial population; followed by *Bacteroidetes*, 19 (S1–S2) to 30–32% (S3 and S4); *Chlorobi*, 10 to 0.4–3% (S3 and S4, respectively); and the remaining bacteria represented <10% of sequences. These phylogenetic groups are representative of those in activated sludge (Johnston et al. 2019; Zhang et al. 2019) and enriched nitrifying cultures (Kapoor et al. 2016; Jeong and Bae 2021).

The 16S-rRNA phylo-taxonomic analysis recognised *Nitrosomona*s sp. (AOB) and *Nitrobacter* sp. (NOB) in the enriched community (see Table S2, Supplementary information). The relative abundance of *Nitrosomonas* sp. increased from 5.2% at the beginning to 5.7% at the end. In terms of *Nitrobacter* sp., they ranged 0.1–0.8% of the total microbial population. Notably, no *Nitrospira* sp., another possible NOB, was found. While these bacteria are typically found in biological wastewater treatment systems, they tend to be more sensitive to environmental conditions (Graham et al. 2007; Knapp and Graham 2007). Further, the prevalence of *Nitrobacter* sp. over *Nitrospira* sp. exposed to higher nitrite levels has been demonstrated (Nogueira and Melo 2006; Nowka et al. 2015).

The final abundances of AOB and NOB species are contingent of the enrichment process; higher proportions of nitrifying bacteria could be grown with long periods of cultivation (Ye et al. 2011; Wang et al. 2019) and fully automatic controlled bioreactors (Yao et al. 2016). In comparison, here, the abundances of AOB ad NOB guilds were within the same order of magnitude to those with similar duration of enrichment (Stadler and Love 2016; Kwon et al. 2019; Jeong and Bae 2021). Additionally, nitrifying activity achieved complete removal of ammonium and nitrate production without nitrite accumulation over the period of enrichment.

Control cultures

**Fig. 1:**
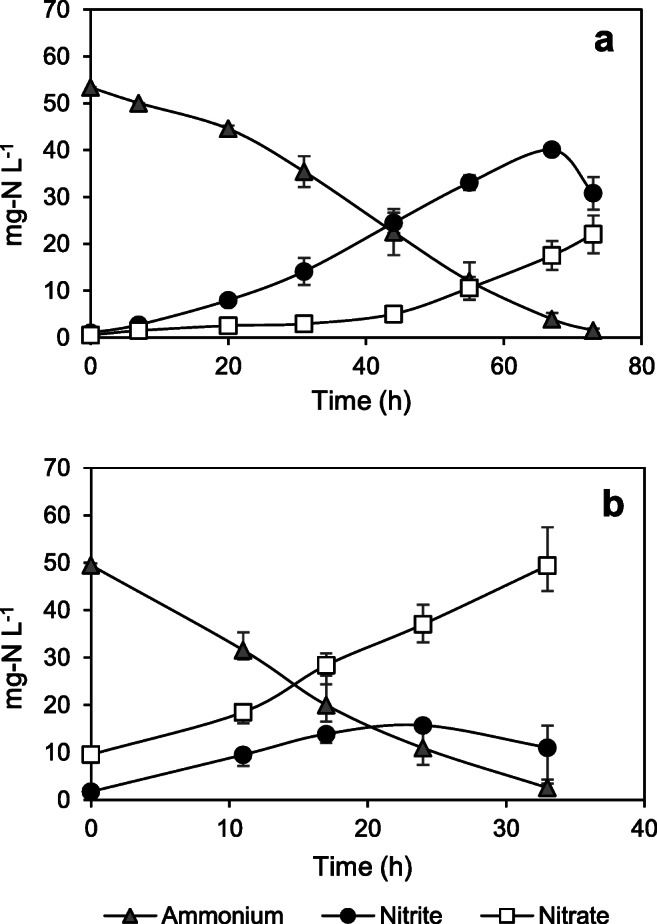
Nitrifying control cultures during the **a** caffeine test and **b** ofloxacin test. Data points show average concentration ± maximum and minimum values

All experimental treatments were inoculated with similar nitrifying consortia, wherein batch reactors absent of the toxicant were used as controls. Figure 1 shows the exemplary performance over time in the controls during the first (CF) and the last (OFX) assays; remaining information is presented in the Supplementary information. All controls achieved microbial nitrification, reducing >90% of the initial ammonium concentration. Moreover, the increase in nitrite and nitrate levels indicated the activities of AOB and NOB, respectively. However, a substantial amount of nitrite accumulated during the incubation, suggesting that the ammonium oxidation rate was higher than the nitrite oxidation rate. At the end of the experiment, nitrite levels were higher in the CF controls (Fig. 1a) than those in the OFX controls (Fig. 1b). This difference could be attributed to modification of culture-rinsing preparations between the two testing groups (as described in the “Materials and methods” section); neither culture was exposed to any contaminant (‘controls’). This effect has been highlighted by other authors (Moussa et al. 2003), and the reduction of oxygen level in the washed biomass could possibly have a detrimental effect on NOB activity (Peng and Zhu 2006).

Experimental conditions were consistent between assays (Table 1). However, temperatures during TCS test were aberrantly +5 °C (Table 1). While the change of temperature within this range can increase the activity of *Nitrosomonas* sp. (AOB species) and *Nitrobacter* sp. (NOB species) by 30 to 40% (Grunditz and Dalhammar 2001), it should be noted that all dose-response assays for each toxicant were conducted simultaneously and compared with controls to minimise collineating factors.

Mass balance of nitrogen based on ammonium consumption, nitrite consumption/production and nitrate production was monitored through the toxicological tests (see Table S1, Supplementary information). Accountability of ammonia transformation to its oxidised products was within ±6% of expectation (mean 0.1%, ±2.6% SD). Besides the slight variations from the chemical analyses, some differences may be due to ammonia volatilisation or assimilation.

Effects of caffeine on nitrification activity

**Fig. 2:**
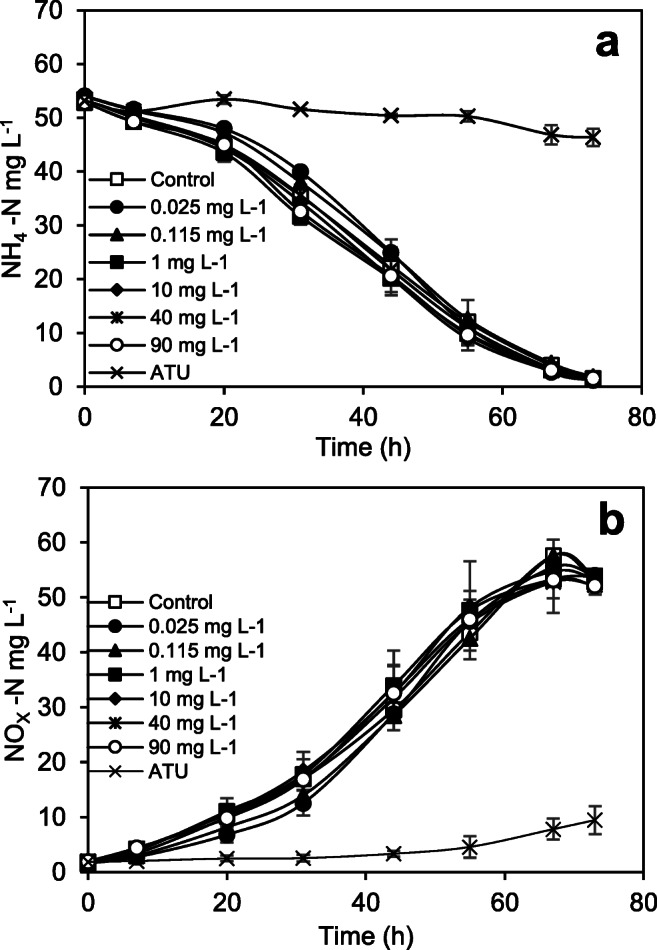
Nitrification profiles of cultures in presence of CF at different concentrations: **a** NH_4_^+^-N and **b** NOx-N (NO_2_^−^ +NO_3_^−^). Data points show average concentration ± maximum and minimum values

Nitrification activity was evaluated by monitoring the concentration of ammonium, and of the oxidation species nitrite and nitrate over time to assess the effects of CF on the nitrifying cultures. Figure 2 shows the nitrification performance of the batch cultures for the different CF concentrations (Table 2), including that of the control culture and the reference inhibitor ATU. At the end of the experiment, 97.4 ± 0.65% of ammonium was consumed in all replicates and the total oxidised compounds (controls and batch reactors spiked with CF) were 51.4 ± 1.0 mg L^−1^ NOx-N with a coefficient of variation less than 2%.

There is scarce information about CF impacts on nitrification; He et al. (2018) suggested nitrification was not impacted during a CF-biodegradation experiment. However, CF can affect other bacteria (Gheorghe et al. 2016). In summary, results indicated that CF did not considerably inhibit nitrification even at the highest concentration at 90 mg L^−1^; neither % inhibition nor EC_50_ was calculated.

Effects of triclosan and DEET on nitrification activity

**Fig. 3:**
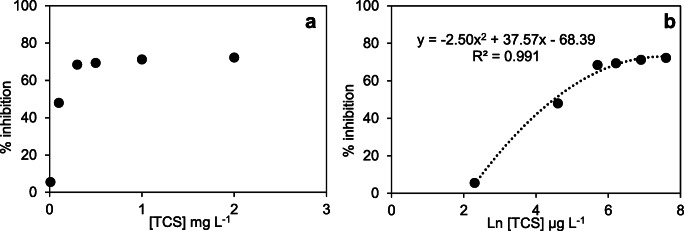
Inhibition level at different concentrations of triclosan

The inhibition (%) at different TCS concentrations was estimated using Eq. (1), and the EC_50_ value was obtained from Fig. 3. There was no observable effect at the lowest concentration of 0.01 mg L^−1^ compared to the control treatments (Fig. 3a); however, a tenfold increase in TCS levels (0.1 mg L^−1^) inhibited nitrification by >50%. Furthermore, inhibition was 72.3% at the highest concentration (2 mg L^−1^). The EC_50_ value calculated for TCS was 89.1 μg L^−1^, with the experimental data (Fig. 3b) adjusted to a second-degree polynomial regression model (*R*^2^ > 0.99).

Similarly, Roh et al. (2009) demonstrated that TCS at 2 mg L^−1^ reduced nitrite production to 70% by using batch reactors with pure AOB cultures as biomass, and Dokianakis et al. (2004) showed that the same concentration of TCS inhibited enriched NOB. However, neither aforementioned study jointly considered AOB–NOB as a community. Furthermore, almost all available data on TCS toxicity from other studies have been obtained from activated sludge. Amariei et al. (2017) reported EC_50_ value of 0.32 ± 0.07 mg L^−1^ with 125 mg (TSS) L^−1^ via a respirometry assay, and via ammonia uptake rates (AUR), Stasinakis et al. (2008) estimated the EC_50_ value of 6.4 mg L^−1^ with 1100–1250 mg (VSS) L^−1^.

Interestingly, all EC_50_ values obtained by the aforementioned studies were higher than the value obtained in the present work. These differences are attributed to the source and concentration of biomass used in the batch test, where lower biomass quantities could lead to lower toxicant tolerances. In this study, the biomass concentration was equivalent to 71 ± 5 mg VSS L^−1^, lower than aforementioned studies. Amariei et al. (2017) demonstrated that the EC_50_ values increased by 17-fold when increasing VSS from 125 to 1000 mg L^−1^. Nevertheless, TCS had a detrimental effect on nitrification, with a considerable activity reduction from 10 to 100 μg L^−1^. These results indicate that TCS may pose a risk on nitrifying bacteria under high peak loadings already reported in WWTPs (Kumar et al. 2010).

**Fig. 4:**
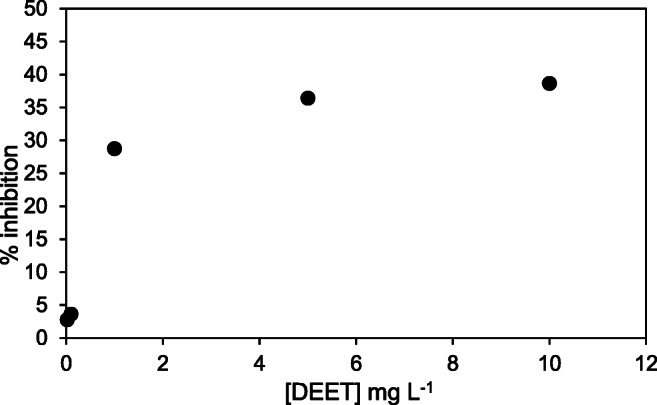
Inhibition percentage at different concentrations of DEET. Maximum nitrification inhibition was below 40%; thus, the regression plot was not performed for EC_50_ estimation

The impact of DEET on nitrification is shown in Fig. 4. DEET had a moderate effect on the nitrifying culture compared with the other PPCPs tested, with 38.7% inhibition at the highest concentration (10 mg L^−1^ DEET). Limited information on the acute toxicity of DEET is available in the literature. Most studies have been carried out on aquatic organisms (Costanzo et al. 2007; Seo et al. 2005), which found lower toxicity (EC_50_ > 100 mg L^−1^) than the concentrations evaluated in the present work. Nevertheless, the batch test with DEET suggested that there is no significant toxicity (inhibition < 3%) at environmental levels reported in the literature (Merel and Snyder 2016; Mohapatra et al. 2016).

Effects of antibiotics on nitrification activity

**Fig. 5:**
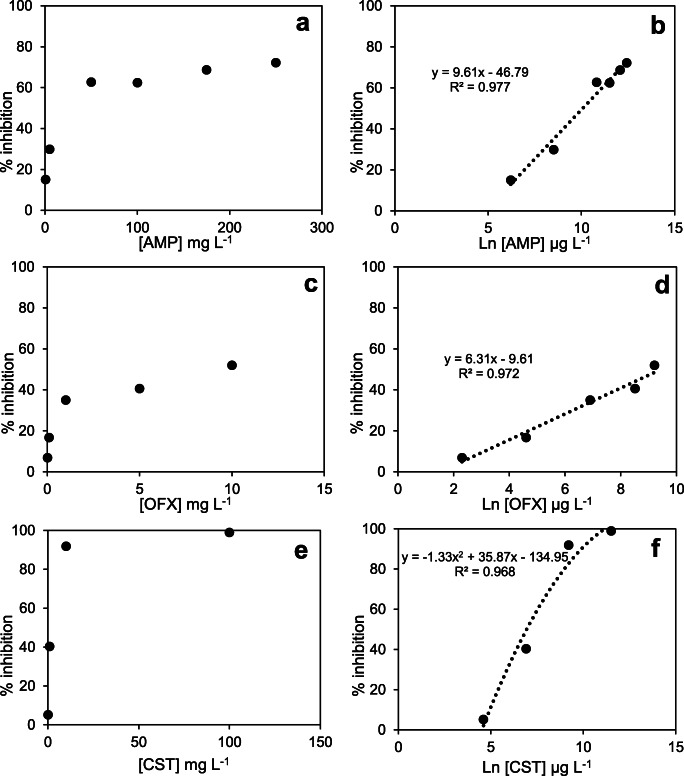
Inhibition level at different antibiotic concentrations: **a**, **b** ampicillin; **c**, **d** ofloxacin; **e**, **f** colistin

The % inhibition results of AMP (ampicillin), OFX (ofloxacin) and CST (colistin) are presented in Fig. 5, along with the EC_50_ estimation plots. In general, all antibiotics had a detrimental effect on nitrification within the range of concentrations tested.

By analysing Fig. 5b, the EC_50_ value of AMP was calculated at 23.7 mg L^−1^, much lower than previous short-term studies with enriched nitrifying bacteria: 250 mg L^−1^ (Gomez et al. 1996) and 50 mg L^−1^ (Ramírez et al. 2020). The enhanced sensitivity of our assay can be attributed to lower biomass and degradation rates of AMP. Yu et al. (2019), with a long-term sequential batch reactor, demonstrated that 30 mg L^−1^ AMP had inhibited nitrification activity by 20 to 32%. Moreover, in this study, activities of ammonia-monooxygenase and nitrite-oxidoreductase declined with the increase in AMP concentration, demonstrating that this antibiotic affects the AOB and NOB.

The plot of OFX % inhibition against the logarithm of the concentration in Fig. 5c was adjusted to a linear regression model (*R*^2^ > 0.97), obtaining an EC_50_ value of 12.7 mg L^−1^ (Fig. 5d). As with DEET, limited data on the acute toxicity of OFX to nitrifying bacteria is found in the literature, and with mixed results. Dorival-García et al. (2013) reported OFX had no inhibitory effect on nitrifying activated sludge at 500 μg L^−1^. When reported, again, EC_50_ values in activated sludge were considerably higher, e.g. 165 mg L^−1^ (Tobajas et al. 2016). However, the EC_50_ value estimated in this study was within the range of OFX levels reported by Dokianakis et al. (2004) for enriched NOB culture: between 6 and 10 mg L^−1^.

Illustrated in Fig. 5f, 50% inhibition by CST was 1 mg L^−1^. This estimation was about tenfold lower than the EC_50_ values reported by Bressan et al. (2013). The changes in the toxicity level may be due to the composition of the CST solution; here, CST sulphate salt (polymyxin E ≥15,000 IU mg^−1^; Sigma Aldrich) was used in the experiments. Bressan et al. (2013) tested two similar commercial CST formulations, one of which contained lactose as vehicle; they reported notable differences in mixed-microbial community tolerance between the two formulations (EC_50_ of 67 mg L^−1^ for CST vs. EC_50_ of 10.8 mg L^−1^ for CST plus lactose), demonstrating that the composition of antibiotics could alter the response of the nitrifying bacteria.

**Fig. 6:**
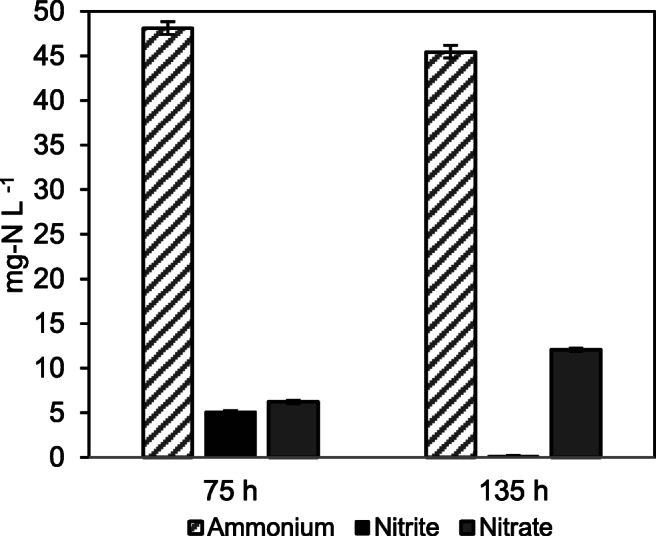
Change of ammonium, nitrite and nitrate levels after the incubation period at concentration of 350 mg L^−1^ colistin

Furthermore, Bressan et al. (2013) highlighted that nitrite oxidation was not affected by CST at their highest concentration (316 mg L^−1^), suggesting that the inhibitory effect of CST was more pronounced on AOB than on NOB. Given that NOB metabolism could be reduced due to lack of substrate availability, we spiked with 1000 mg L^−1^ sodium nitrite (NaNO_2_) stock solution at the end of the incubation period to evaluate the response of NOB under the highest CST concentration (350 mg L^−1^) and corroborate the results reported by Bressan et al. (2013). The considerable reduction of nitrite levels (~98%) and the increase in nitrate levels to 93.8% demonstrated that CST had a low impact on NOB (Fig. 6). Moreover, the ammonium consumption remained low (<5.5%), showing the persistent inhibition of CST on AOB metabolism.

In general, most of the PPCPs tested in this study had an impact on the performance of nitrifiers with the exception of CF. The measurement of ammonium and oxidation products directly reflected the effects of each PPCP on AOB/NOB guilds compared to the control treatments. However, remarkable differences were observed with previous inhibition studies. Further research is required to determine how the concentration of nitrifying biomass can affect the toxicity tolerance against these contaminants. Additional experiments should be performed to evaluate other exposure scenarios, such as biomass acclimation, where the microbial communities could show higher capacity to withstand a wide range of chemicals and exhibit possible synergistic effects of PPCP mixtures on nitrification activity.

In summary, the inhibition capacity (EC_50_) of TCS, AMP, OFX and CST in short exposure batch tests with nitrifying bacteria was 89.1 μg L^−1^, 23.7 mg L^−1^, 12.7 mg L^−1^ and 1 mg L^−1^, respectively. The maximum inhibition in the presence of DEET was close to 40% at 10 mg L^−1^, whereas no remarkable effect was observed for CF in concentrations up to 90 mg L^-1^. Among the PPCPs tested, TCS exhibited a more pronounced effect on nitrification activity at the concentrations above 0.01 mg L^−1^. Although the TCS levels reported in the environment are rarely near the EC_50_ values estimated in this study, this antimicrobial agent is commonly detected in WWTPs worldwide at higher concentrations compared to other PPCPs of concern (Tran et al. 2018). The data suggested that TCS is still widely consumed despite the efforts of governmental agencies to restrict its application in numerous household and personal care products (Bedoux et al. 2012). Therefore, TCS usage should be revised to control its excessive consumption and further disposal in the sewage that may lead to its accumulation at higher concentrations in the environment, thereby posing a risk to non-target microorganisms.

The results of acute toxicity analysis indicated that the levels of AMP and OFX detected in the environment were too low to inhibit nitrifier metabolism, considering that antibiotics often occur at concentrations from ng L^−1^ to μg L^−1^ (Kümmerer 2009). Moreover, further research is necessary to investigate the occurrence and fate of CST in different habitats and evaluate the risks of CST concentrations in the environment.

## Conclusions

The toxicity of the most common PPCPs was investigated using short-term nitrification inhibition assays. Based on the 16S rRNA gene sequencing and analysis, *Nitrosomonas* sp. belonging to AOB and *Nitrobacter* sp., a common NOB, were identified in the nitrifying biomass along with other microbial groups typically found in activated sludge. The experimental results of acute toxicity to enriched nitrifying bacteria obtained in this study suggested that the most toxic chemical was TCS compared to the other 5 PPCPs, showing the lowest EC_50_ value of 89.1 μg L^−1^. With regard to the antibiotics, CST exerted the highest toxicity to overall nitrification (EC_50_ value of 1 mg L^−1^), with a more pronounced inhibition on AOB activity than on NOB activity. Results showed that the EC_50_ values estimated for AMP (23.7 mg L^−1^) and OFX (12.7 mg L^−1^) were considerably higher than environmentally relevant levels. CF had no remarkable inhibitory effects on nitrification performance. In the case of the insect repellent, DEET exerted partial inhibitory effects on nitrifying bacteria below 40% at the highest concentration (10 mg L^−1^).

## Supplementary information

ESM 1 (DOCX 567 kb)

## Data Availability

The datasets used and/or analysed during the current study are available from the corresponding author Dr. Charles W. Knapp (charles.knapp@strath.ac.uk) on reasonable request.
