# Supplementary material for: Pharmaceuticals and personal care products’ (PPCPs) impact on enriched nitrifying cultures
Source: Environ Sci Pollut Res Int. 2021 Jun 24;28(43):60968–80. doi: 10.1007/s11356-021-14696-7 (PMC8580922; doi:10.1007/s11356-021-14696-7)
Supplement: Supplementary file 1 — (DOCX 567 kb) [file 11356_2021_14696_MOESM1_ESM.docx]

Pharmaceuticals and Personal Care Products (PPCPs) Impacts’ on Enriched Nitrifying Cultures

Carla Lopez^1^, Mac-Anthony Nnorom^1^, Yiu Fai Tsang^2^, Charles W. Knapp^1^

1 – Centre for Water, Environment, Sustainability & Public Health; Department of Civil & Environmental Engineering; University of Strathclyde, Glasgow, United Kingdom G1 1XJ

2 – Department of Science and Environmental Studies, The Education University of Hong Kong, Hong Kong

**Table S1.** Nitrogen mass balance

| **Substance** | **Concentrations (mg L^-1^)** | **DNH_4_^+^-N**  **(mg-N L^-1^)** | **DNO_2_^-^-N**  **(mg-N L^-1^)** | **DNO_3_^-^-N**  **(mg-N L^-1^)** | **Difference (%)*** |
| --- | --- | --- | --- | --- | --- |
| CF | 0 | 51.88 | 29.80 | 21.53 | 1.05 |
|  | 0.025 | 52.92 | 20.1 | 32.30 | 0.97 |
|  | 0.115 | 52.12 | 30.91 | 21.21 | 0.07 |
|  | 1 | 51.52 | 28.90 | 23.09 | -0.91 |
|  | 10 | 52.10 | 26.09 | 25.32 | 1.33 |
|  | 40 | 51.92 | 27.22 | 23.04 | 3.19 |
|  | 90 | 51.41 | 24.92 | 25.28 | 2.36 |
| TCS | 0 | 47.46 | 5.56 | 41.46 | 0.93 |
|  | 0.01 | 43.59 | 7.24 | 37.15 | -1.85 |
|  | 0.1 | 24.06 | 6.59 | 17.88 | -1.65 |
|  | 0.3 | 14.34 | 6.95 | 7.88 | -3.41 |
|  | 0.5 | 14.67 | 6.92 | 7.45 | 2.03 |
|  | 1 | 13.40 | 5.21 | 8.30 | -0.89 |
|  | 2 | 13.24 | 6.14 | 6.90 | 1.54 |
| DEET | 0 | 47.70 | 6.36 | 41.77 | -0.91 |
|  | 0.02 | 44.82 | 8.94 | 37.86 | -4.43 |
|  | 0.1 | 47.03 | 7.41 | 38.47 | 1.36 |
|  | 1 | 35.24 | 14.71 | 19.60 | 2.65 |
|  | 5 | 30.69 | 12.93 | 17.68 | 0.28 |
|  | 10 | 29.49 | 13.80 | 15.73 | -0.14 |
| AMP | 0 | 51.77 | 25.76 | 25.88 | 0.25 |
|  | 0.5 | 43.94 | 21.89 | 22.00 | 0.11 |
|  | 5 | 36.33 | 27.65 | 8.57 | 0.28 |
|  | 50 | 19.43 | 13.02 | 6.24 | 0.88 |
|  | 100 | 19.57 | 12.82 | 6.58 | 0.85 |
|  | 175 | 15.89 | 10.49 | 5.70 | -1.87 |
|  | 250 | 14.41 | 9.87 | 4.49 | 0.33 |
| CST | 0 | 47.10 | 12.53 | 33.40 | 2.48 |
|  | 0.1 | 44.83 | 13.50 | 30.08 | 2.77 |
|  | 1 | 28.18 | 8.98 | 18.42 | 2.77 |
|  | 10 | 7.61 | -0.59 | 4.32 | ** |
|  | 100 | 3.49 | -0.49 | -0.02 | ** |
|  | 350 | 2.18 | -0.51 | 0.24 | ** |
| OFX | 0 | 46.90 | 9.27 | 39.82 | -4.67 |
|  | 0.01 | 43.88 | 9.95 | 35.81 | -4.28 |
|  | 0.1 | 41.37 | 13.36 | 27.55 | 1.12 |
|  | 1 | 30.88 | 12.77 | 19.13 | -3.34 |
|  | 5 | 31.27 | 9.24 | 19.94 | 6.66 |
|  | 10 | 22.36 | 9.48 | 14.10 | -5.48 |

* Difference estimated as (DNH_4_^+^-N - DNO_X_-N)/ DNH_4_^+^-N*100 ** Value excluded due to low concentration of oxidising species.

**16S-rRNA gene sequencing and taxonomy classification**

The 16S-rRNA gene sequences analysis (V3 and V4 regions) was performed with QIIME2 (version 2021.2) with similar workflow described by Al Ali et al. (2020). Briefly, the raw data were paired-end read merged and trimmed to remove primers, adapters sequences and low quality reads, following by denoising, chimera removal, and de-replication. Remaining sequences were classified taxonomically in term of amplicon sequence variant (ASV) using GreenGenes 16S-rRNA gene database available in the software website (www.qiime2.com). The results at phylum level are summarized in the Figure S1.


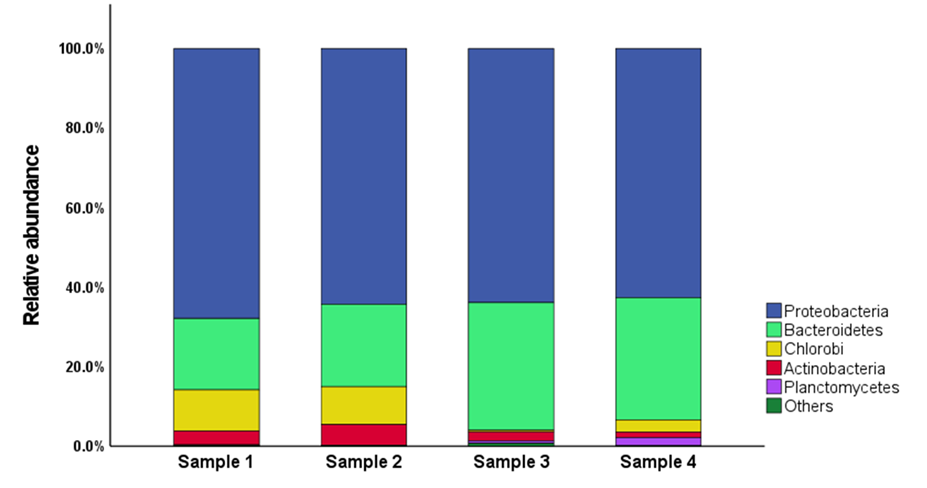


**Fig. S1.** Relative abundance of the total microbial community at phylum level.

**Table S2.** Relative abundance (total bacterial population) of nitrifying species

| Nitrifying species | Average Abundance [%] | | | |
| --- | --- | --- | --- | --- |
|  | Sample 1 | Sample 2 | Sample 3 | Sample 4 |
| k__Bacteria; p__Proteobacteria; c__Betaproteobacteria;o__  Nitrosomonadales;f__Nitrosomonadaceae | 4.35 | 4.23 | 5.76 | 5.71 |
| k__Bacteria; p__Proteobacteria; c__Betaproteobacteria;o__  Nitrosomonadales;f__Nitrosomonadaceae;g__Nitrosomonas; | 0.92 | 0.86 |  |  |
| k__Bacteria; p__Proteobacteria; c__Alphaproteobacteria;o__  Rhizobiales;f__Bradyrhizobiaceae;g__Nitrobacter;s__ | 0.83 | 0.79 | 0.14 | 0.36 |


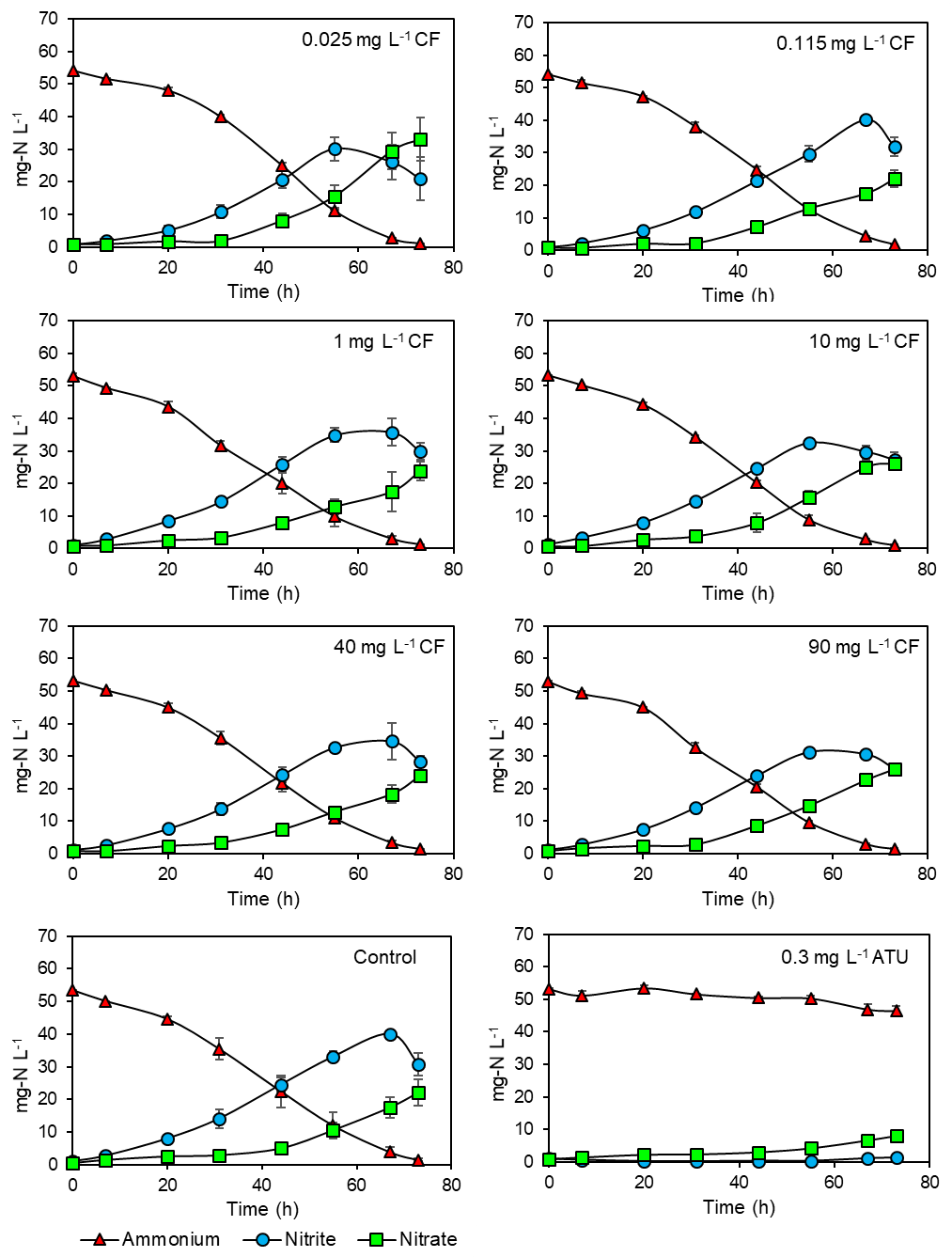


**Fig. S2**. Nitrification profile of nitrifying cultures in presence of caffeine (CF). Data points show average concentration ± highest and lowest values


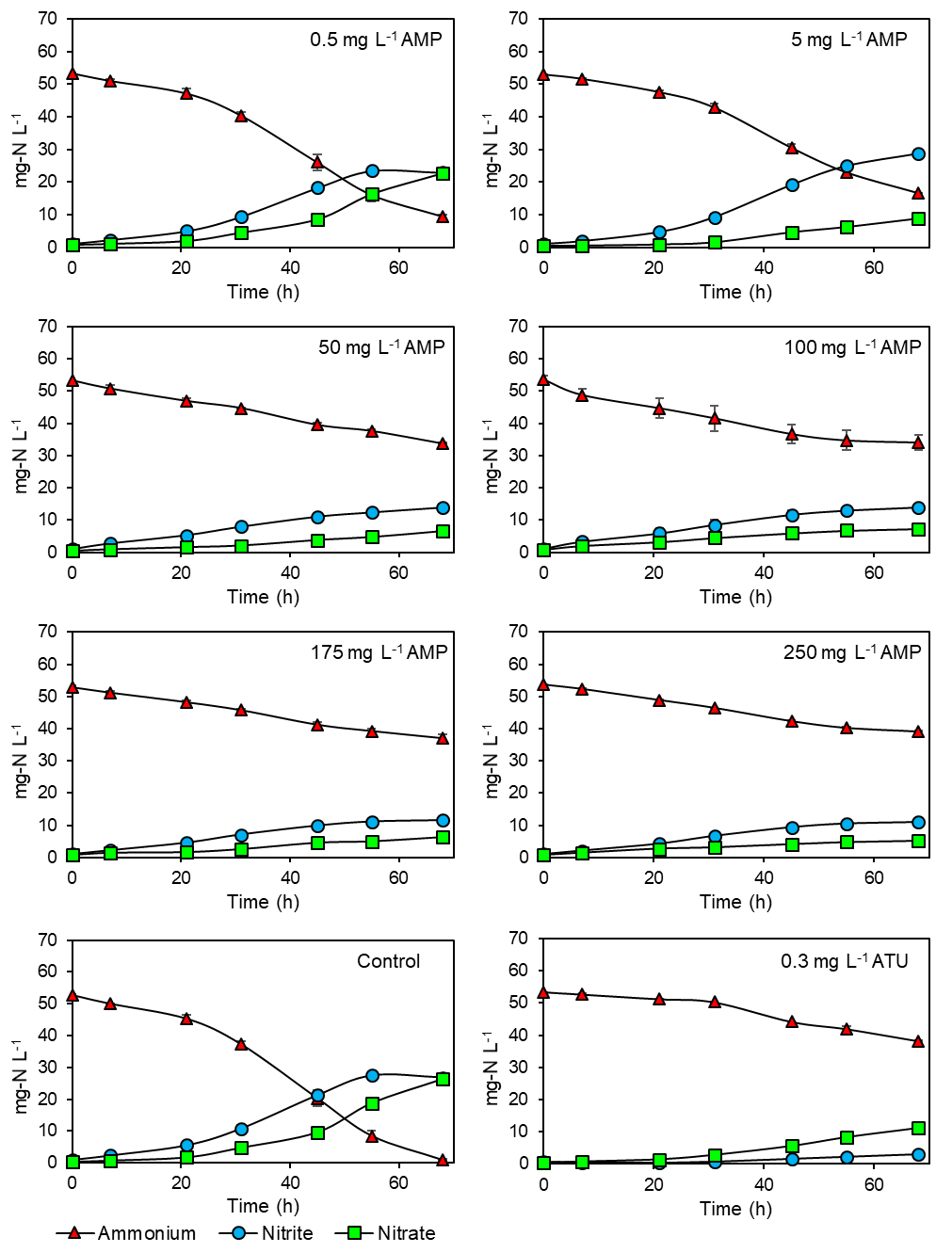


**Fig. S3**. Nitrification profile of nitrifying cultures in presence of ampicillin (AMP). Data points show average concentration ± highest and lowest values


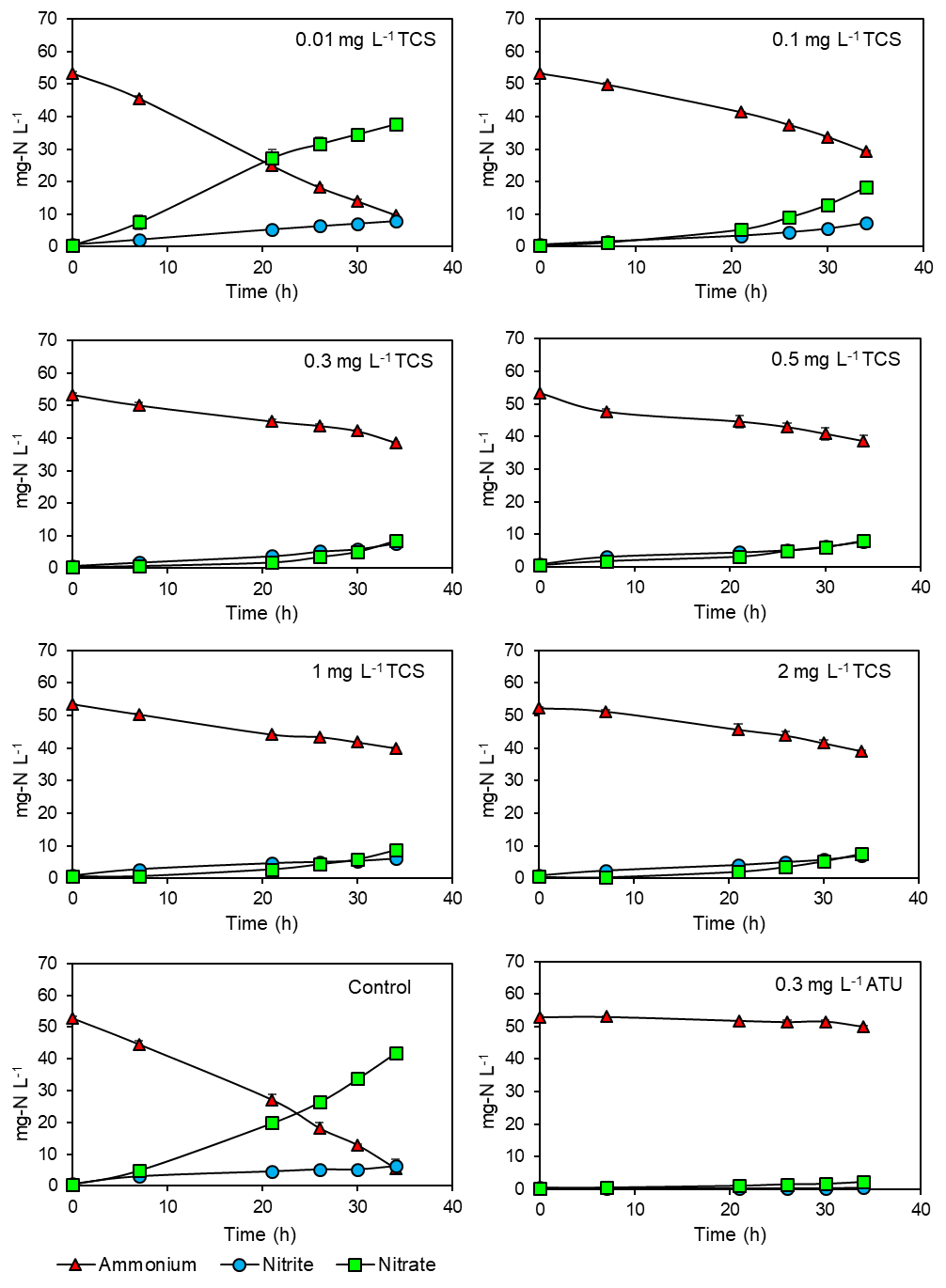


**Fig. S4**. Nitrification profile of nitrifying cultures in presence of triclosan (TCS). Data points show average concentration ± highest and lowest values


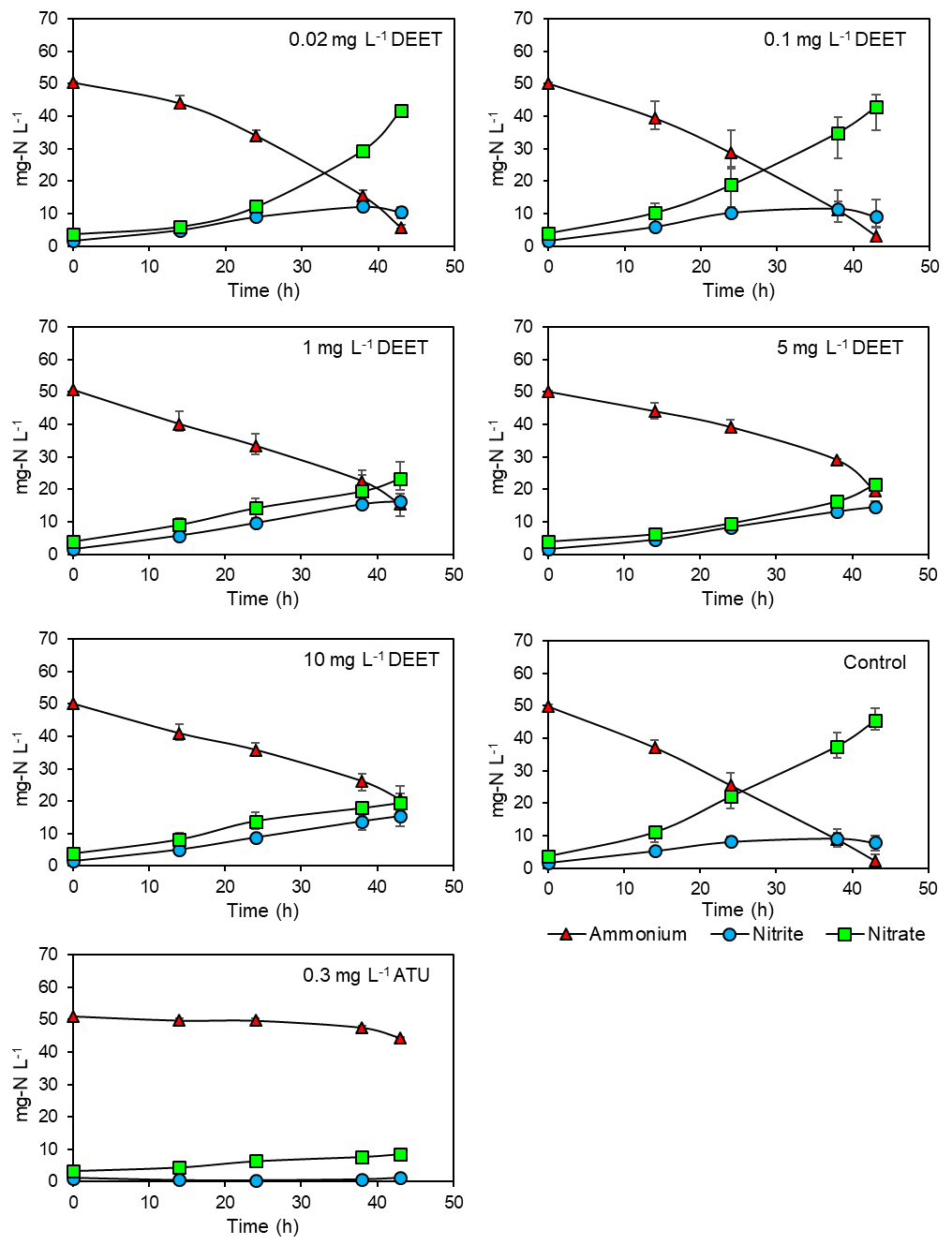


**Fig. S5**. Nitrification profile of nitrifying cultures in presence of DEET. Data points show average concentration ± highest and lowest values


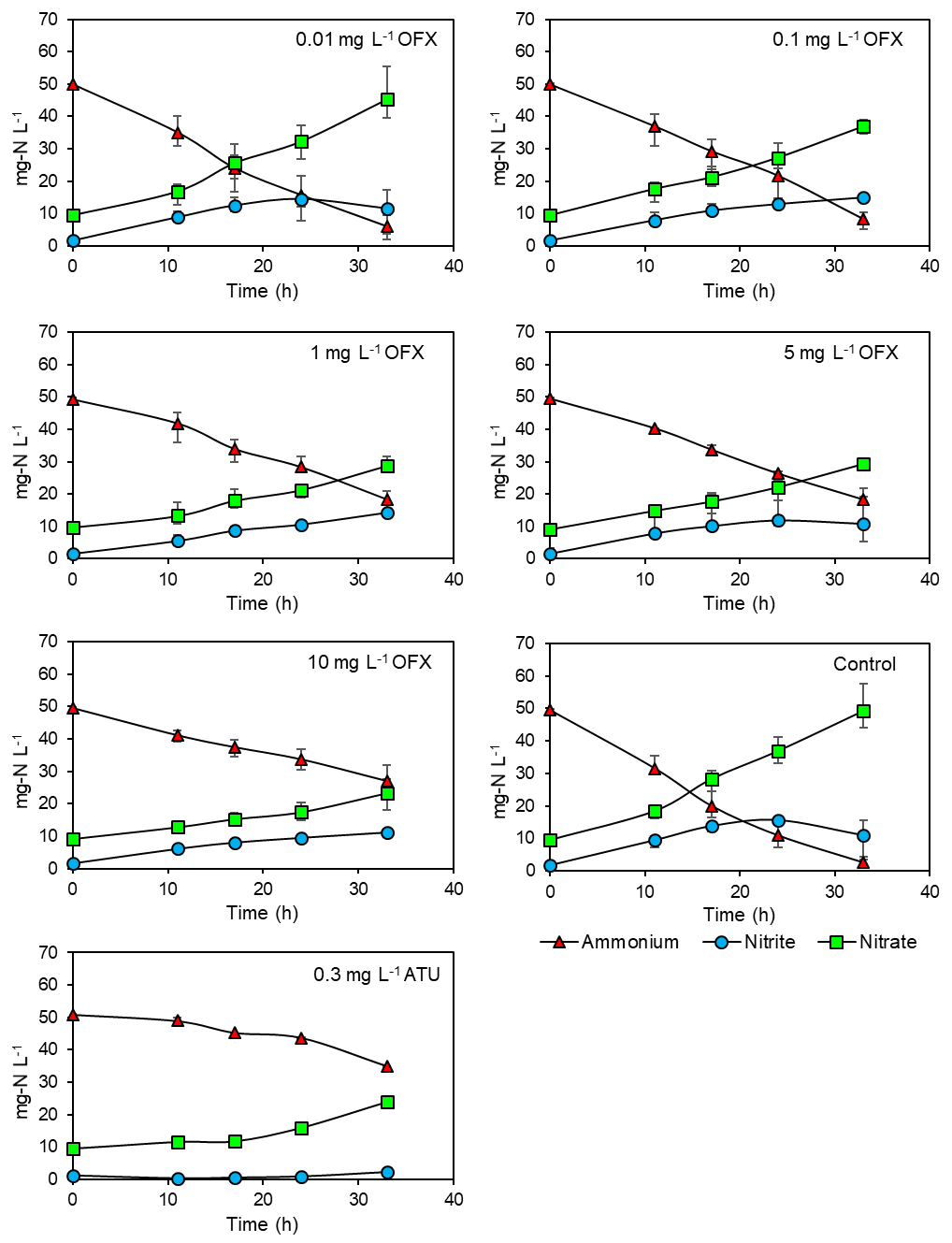


**Fig. S6**. Nitrification profile of nitrifying cultures in presence of ofloxacin (OFX). Data points show average concentration ± highest and lowest values


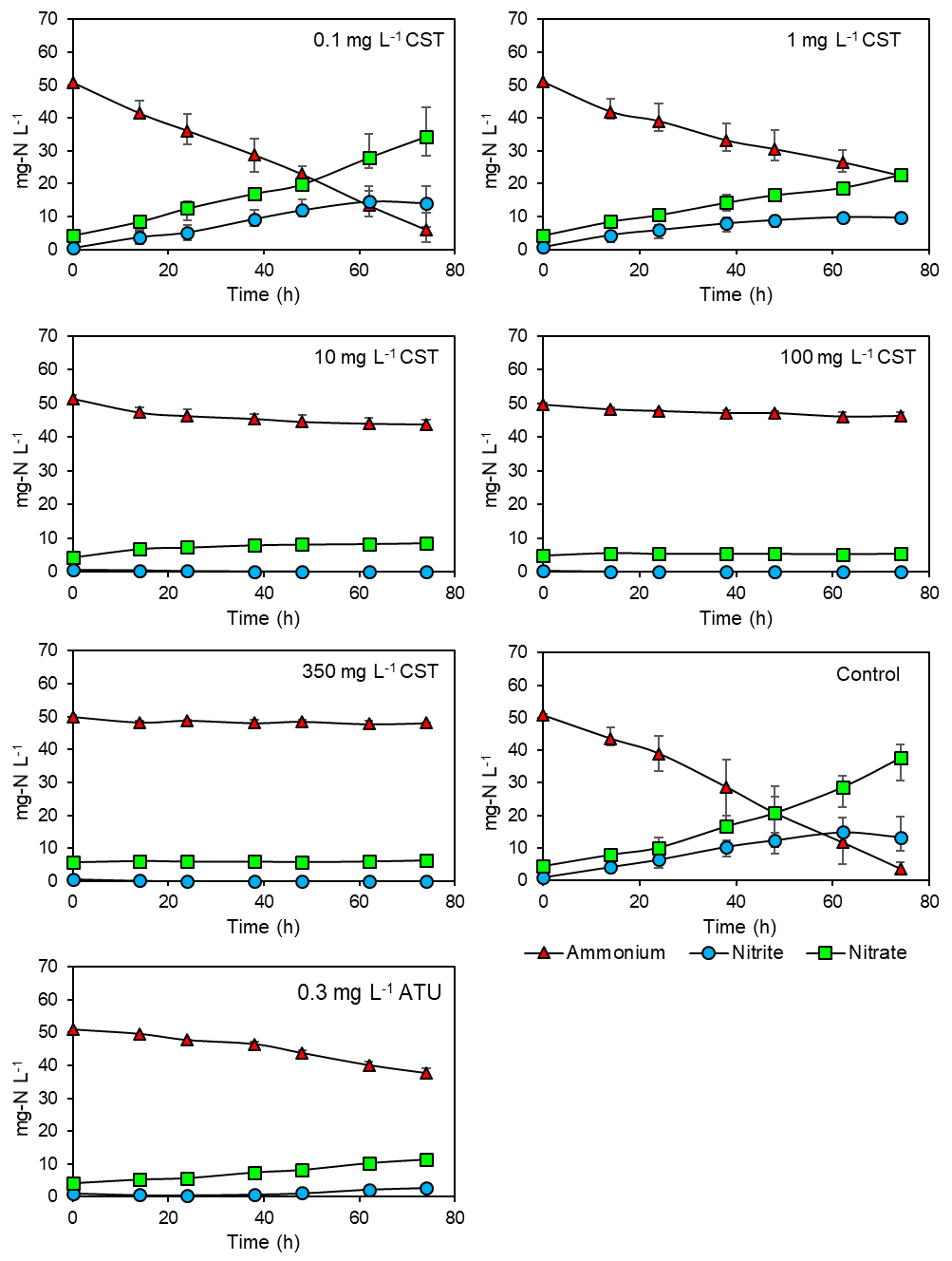


**Fig. S7**. Nitrification profile of nitrifying cultures in presence of colistin (CST). Data points show average concentration ± highest and lowest values

References

Al Ali AA, Naddeo V, Hasan SW, Yousef AF (2020) Correlation between bacterial community structure and performance efficiency of a full-scale wastewater treatment plant. J Water Process Eng. 37: 101472. https://doi.org/10.1016/j.jwpe.2020.101472
